# Prevention of tick bites: an evaluation of a smartphone app

**DOI:** 10.1186/s12879-017-2836-4

**Published:** 2017-12-04

**Authors:** L. Antonise-Kamp, D. J. M. A. Beaujean, R. Crutzen, J. E. van Steenbergen, D. Ruwaard

**Affiliations:** 1 National Institute for Public Health and the Environment, Centre for Infectious Disease Control, P.O. Box 1, 3720 BA Bilthoven, the Netherlands; 2 Faculty of Health, Medicine and Life Sciences, Care and Public Health Research Institute (CAPHRI), Department of Health Promotion, Maastricht University, P.O. Box 616, 6200 MD Maastricht, the Netherlands; 3 Centre for Infectious Diseases, Leiden University Medical Centre, P.O. Box 9600, 2300 RC Leiden, the Netherlands

**Keywords:** Lyme disease, public health, *Borrelia*, ticks, mobile application, smartphone, Educational interventions

## Abstract

**Background:**

Lyme borreliosis (LB) is the most common reported tick-borne infection in Europe, and involves transmission of *Borrelia* by ticks. As long as a vaccine is not available and effective measures for controlling tick populations are insufficient, LB control is focused on preventive measures to avoid tick bites. To inform citizens about the risk of ticks, motivate them to check for tick bites, and encourage them to remove any attached tick as quickly as possible, a mobile app called ‘Tekenbeet’ (Dutch for ‘tick bite’) was developed and released. The aim of this study was to evaluate the usage and user satisfaction of the ‘Tekenbeet’ app and to investigate whether it affects users’ knowledge, perceived severity, perceived susceptibility, self-efficacy, response efficacy, current behavior and intention to comply with preventive measures.

**Methods:**

Usage of the app was evaluated with data obtained from Google Analytics. A survey among the Dutch general adult population with two data collection periods evaluated the usage, user satisfaction and its influence on abovementioned outcomes.

**Results:**

Data obtained from Google Analytics showed the app was downloaded almost 40,000 in the 20 months following the launch. The ‘tick radar’ and ‘tick diary’ screens were viewed most often. In addition, a total of 554 respondents completed an online survey. The mean user satisfaction score was 7.44 (on a scale of 1–10) and 90.9% of respondents would recommend the app to others. On average, survey respondents who downloaded the app (*n* = 243) recorded significantly more often higher knowledge scores (OR 3.37; 95% CI 2.02–5.09) and had a higher intention to comply with preventive measures (OR 2.47; 95% CI 1.22–5.85) compared to respondents who did not download the app (*n* = 311).

**Conclusions:**

The ‘Tekenbeet’ app is a frequently used and well-appreciated educational tool to increase public knowledge of ticks and tick bites. It also helps to improve the user’s intention to apply preventive measures. The use of smartphones and apps is now commonplace in the Netherlands; the ‘Tekenbeet’ app feeds into this trend and thereby offers a modern day alternative to established formats such as an information leaflet and information provision on the Internet.

**Electronic supplementary material:**

The online version of this article (10.1186/s12879-017-2836-4) contains supplementary material, which is available to authorized users.

## Background

Lyme Borreliosis (LB) is caused by different *Borrelia* species, which in Europe are transmitted by the tick *Ixodes ricinus*. The most common clinical symptom of LB is erythema migrans (EM), a characteristic rash expanding from the site of the tick bite, which may appear several days to weeks following infection, and is sometimes accompanied by systemic flu-like symptoms. Progressive LB can develop into a multi-systemic disease with skin, neurological, cardiac and musculoskeletal manifestations [1]. In Western Europe there have been a multitude of ecological changes over the last decades; a huge increase in afforestation, a much stronger growth of vegetation due to increased use of fertilizing agents and a rise in CO_2_ concentrations, more rainfall and increased humidity levels [2]. This has resulted in improved living conditions for ticks. In the Netherlands, there has been a continuing and strong increase in general practitioner consultations for both tick bites and EM, from 191 per 100,000 inhabitants in 1994 to 564 in 2009 [3–5]. More than one million people (8% of the total Dutch population) obtain a tick bite each year, making LB a serious threat to public health [6, 7]. Hence there is a genuine need to inform and educate the public about the risks associated with tick bites, especially since not only LB is transmitted by ticks, but also the first case of tick-borne encephalitis recently has been diagnosed in the Netherlands [8].

At present, a vaccine is not available and effective measures for controlling tick populations are not sufficient or are still in the experimental phase. Therefore, a reduction in LB incidence should be focused on preventative behavioral measures for avoidance of tick bites and hence achieving prevention of *Borrelia* transmission [9–11]. These behavioral measures consist of the use of protective clothing and/or using repellants initially, then subsequently checking for, and immediately removing any attached ticks after spending time in tick habitats. Conducting such behavioral measures has been found to be an effective and cost-efficient method for LB prevention [12]. The Dutch National Institute for Public Health and the Environment (RIVM) provides information on public health topics for professionals and the general public. This includes a national guideline on the prevention and control of LB for professionals and several educational materials for the general public [13]. An annual media campaign called ‘de week van de teek’ (‘Tick-bite Prevention Week’) aims to inform the general public about the health risks associated with tick bites at the start of the tick season.

Current evidence on the relationship between knowledge on ticks and the application of preventative behavior is conflicting. Gould et al. have found a significant positive correlation between knowledge and protective behavior [14]. However, research by Corapi, in areas where LB is endemic, demonstrated that many people fail to show behavior changes aimed at reducing their risk of LB infection, despite having adequate knowledge about its symptoms and transmission [14, 15]. This suggests that (acquired) knowledge may influence the intention to change behavior, but it does not necessarily guarantee a sustained change in habit. Moreover, a message is best received if it is tailor-made and presented at the optimal moment [16]. One of the tools that can possibly meet this requirement is mobile health technology (mHealth). Traditionally, health information has been presented in paper format, via leaflets or posters. These are not readily accessible in times of need, for example if a person is hiking in a tick-rich environment. mHealth can make this information more accessible, by making it available on a smartphone, something people have with them most of the time [16, 17]. This study aimed to evaluate a dedicated tick-related smartphone application. Questions to be answered included: what is the usage and user satisfaction of the app and what are the effects of this kind of mHealth technology on user knowledge, perceived severity, perceived susceptibility, self-efficacy, response efficacy, and intention to comply with measures to prevent tick bites and LB?

### The ‘Tekenbeet’ mobile app

**Fig. 1:**
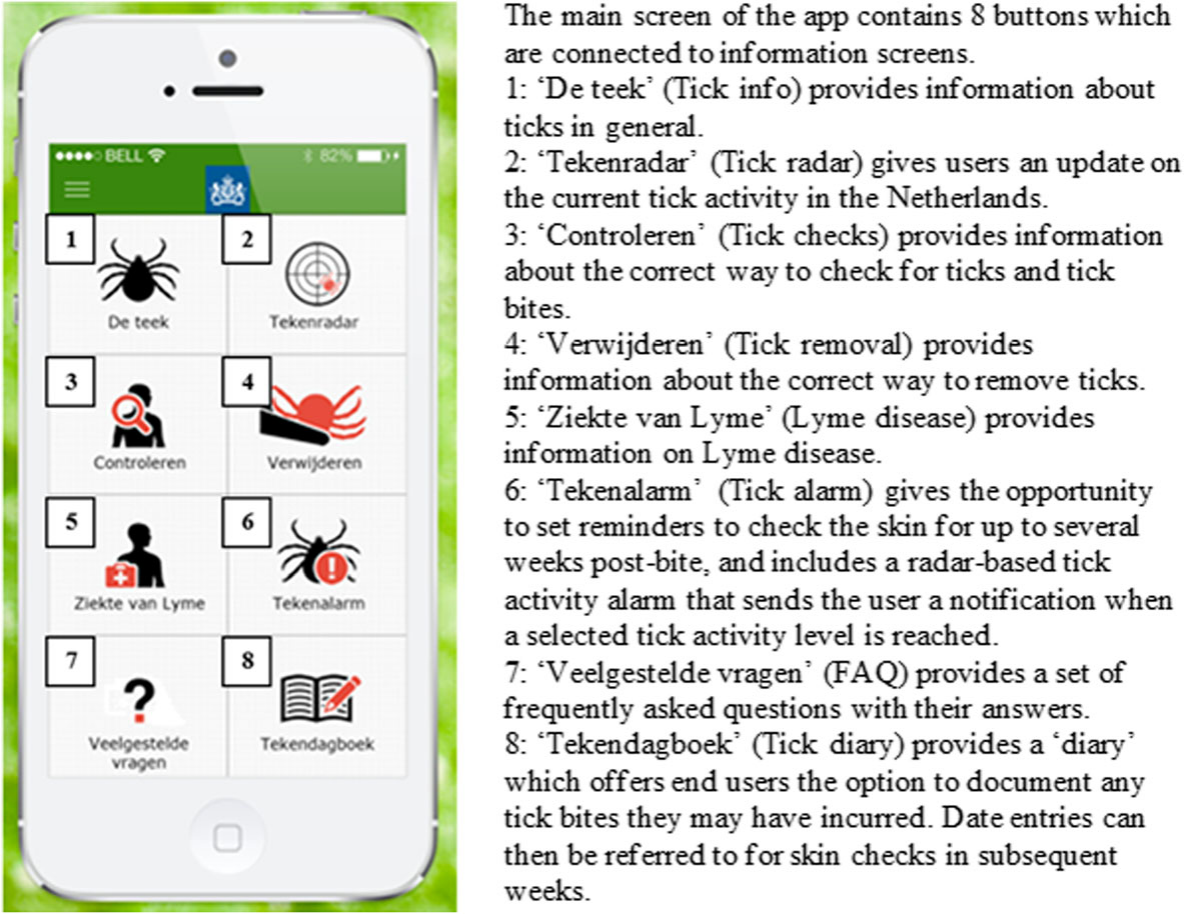
An overview of the functionality of the ‘Tekenbeet’ app

We have previously studied how to design an electronic health intervention for prevention of tick bites and LB by using end-user profiling and a value-based design [17, 18]. This previous study resulted in a set of requirements that not only specify what the technology should do (functionalities), but also how it should be implemented. These requirements were best suited to a mobile app. The app called ‘Tekenbeet’ (Dutch for ‘tick bite’) is a smartphone application aimed at supporting the public in dealing with exposure to ticks and tick bites. The app is aimed at the prevention of tick bites and LB by providing relevant information, including instructions on the correct way to check for, and remove ticks; and providing intelligence on tick activity in the Netherlands (‘tick radar’). Furthermore, the app also offers the option for users to document any tick bites sustained, allows reminders to be set to check one’s skin for up to several weeks post-bite, and includes a tick activity alarm, based on ‘tick radar’, that sends the user a notification when a selected tick activity level is reached. A more detailed explanation of the app’s functionality can be found in Fig. 1.

The freely available Android-based ‘Tekenbeet’ app was developed by RIVM and available from April 2014. It was achieved in partnership with several stakeholders such as national nature and recreational organizations, Association of Community Health Services Netherlands, Scouting Netherlands, and several universities. Originally, the app was only available for Android phones since this was the most popular operating system in the Netherlands. Due to popular demand, in July 2014 the app became available for iOS as well. The app can be downloaded for Android smartphones at: https://play.google.com/store/apps/details?id=nl.ddt.tick&hl=nl; and for iPhones at: https://itunes.apple.com/nl/app/tekenbeet/id894584051?mt=8.

**Fig. 2:**
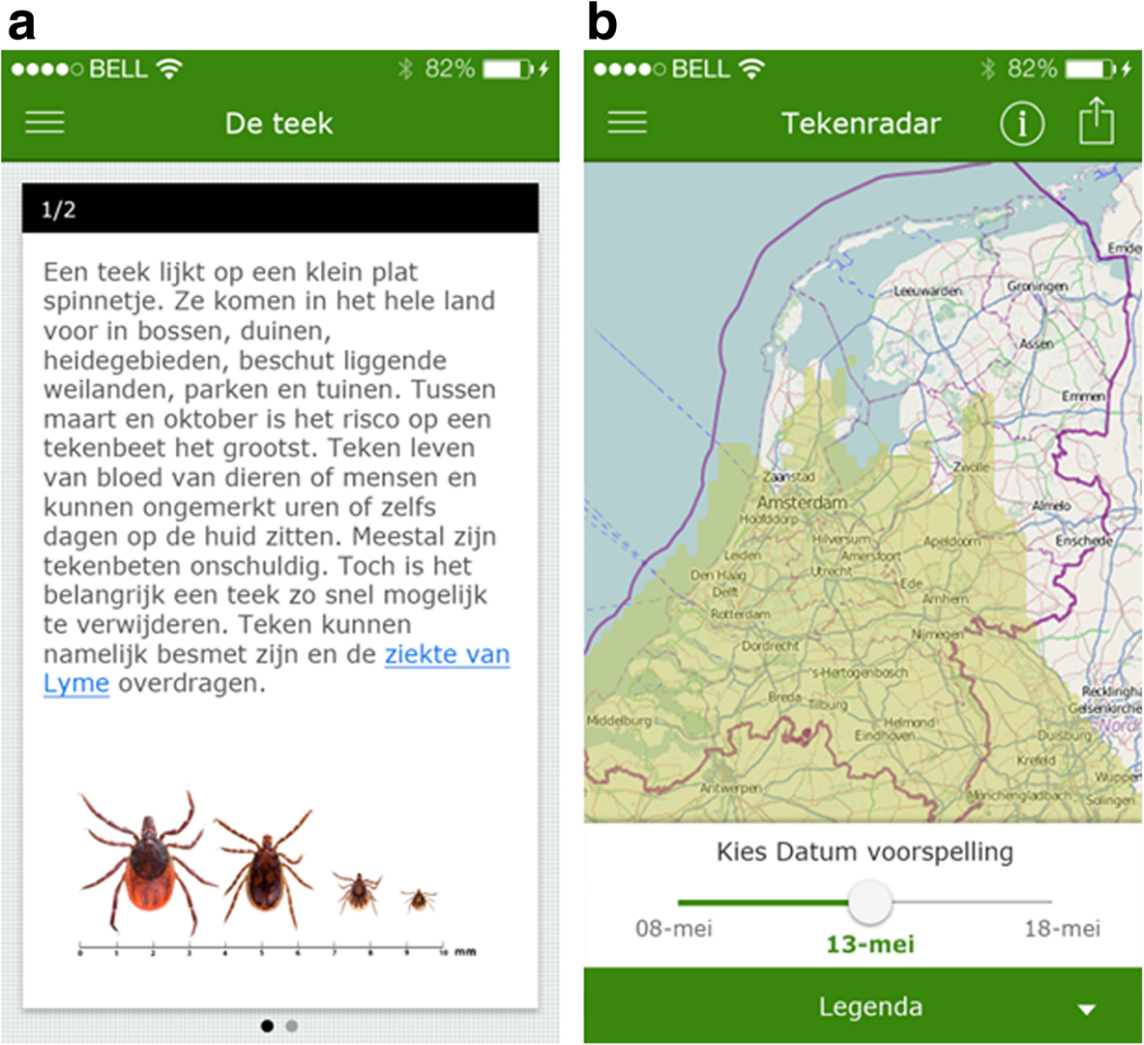
Examples of screenshots of the ‘Tekenbeet’ app. **a** Tick information screen about the appearance of ticks, their habitat, and how they can cause Lyme disease. **b** Tick radar screens show the current activity of ticks (nymphs) in the Netherlands and a ten day forecast. Light green implies a small risk for tick bites and white a minimal risk

On-the-spot Internet access is not required for most components of the app, apart from the ‘tick radar’, which assures that most of the app’s functionality is available anywhere. Screenshots of the app are shown below. Figure 2a and b show the screens with tick information and the ‘tick radar’ screen, respectively.

## Methods

### Pilot test

Before the final version of the app became available in the app stores, a pilot test was carried out among 10 participants. Test users included an expert tick biologist (*n* = 1) and experts in communication (*n* = 2), whilst others were member of the general public (*n* = 7). While being observed by two researchers, each participant received a list of tasks to perform with the app, such as ‘You are going for a walk in the woods tomorrow and would like to know the risk of a tick bite. Find out the risk level’. While performing the tasks using the app, participants were encouraged to give live critical feedback (a so-called think aloud procedure). Besides these tasks, more general questions were asked such as ‘Which functionalities do you expect to find in this app?’, and ‘When would you like to use this app?’. This resulted in a list of comments, addressed before the final app version was finalized, such as ‘please show more pictures of ticks and tick bites’, which was solved by adding more pictures, and ‘it would be nice to view the video in full screen mode’, which was solved by creating a full screen video option in the app.

### Google analytics to evaluate usage of the ‘Tekenbeet’ app

Usage statistics such as number of downloads, popularity of the different screen types, and amount of returning users were recorded using Google Analytics [19] and provided app data at the aggregate level.

### Survey to evaluate usage, user satisfaction, and the effect of the ‘Tekenbeet’ app

#### Study design and participant recruitment

A survey with two questionnaires, one per time point, was carried out in order to evaluate usage, user satisfaction and the effect of the mobile ‘Tekenbeet’ app. According to Dutch law, this general Internet-based survey involving healthy volunteers from the general population requires no formal medical ethical approval.

During the first data collection period (Additional file 1: Questionnaire 1), respondents were recruited through several channels. To fill out the questionnaire, it was not necessary for respondents to have downloaded the app or to even be aware of its availability; anyone willing to participate could do so. A call for participants for this study was posted on the RIVM website (https://www.rivm.nl/tekenbeet), which attracts around 220,000 unique visitors annually, who are most likely interested in information on ticks and LB. Therefore this group of visitors represents a large part of the target group on which this mobile app is focused, and from which it is important to get relevant results. In addition, several other external websites from our stakeholders such as national nature organizations, Scouting Netherlands and the Foundation Tick Bite Diseases advertised this call for participants. Furthermore, several organizations included a call in their subscriber newsletters. After filling out questionnaire 1, respondents who were willing to fill out a second questionnaire (Additional file 1: Questionnaire 2) a few weeks later, could leave their e-mail address. To increase response rates, participants could win one of 50 gift certificates with a value of 10 Euro if they filled out both questionnaires.

The first questionnaire (Additional file 1: Questionnaire 1) could be filled out from 18th July till 1st October 2014; the second questionnaire (Additional file 1: Questionnaire 2) from 6th till 30th October 2014. The first questionnaire had a much longer inclusion time compared to the second, which was due to the inclusion methods. During the first questionnaire, respondents were passively recruited. During the second questionnaire, respondents who indicated that they were willing to participate left their e-mail address at the end of the first questionnaire and could therefore be contacted directly. It was important to collect the data before the end of the tick season (end of October) to ensure the content of the questionnaire was still relevant. It was not the goal to randomly allocate the respondents over the two groups (i.e. those who did and those who did not download the app), since it was impossible to force respondents to refrain from downloading the app as it is publicly available. Therefore respondents were assigned to a group based on their willingness to download the app or not. After agreeing to fill out the questionnaire, respondents were asked if they were willing to download the app if they did not already have the app. Respondents who already had the app or were voluntarily willing to download the app were included in the app user group (the group of people who already downloaded the app or did so in the beginning of the first questionnaire). Respondents who were not willing to download the app or did not have a smartphone were included in the non-app user group (the group of people who did not download the app).

#### Questionnaires

The questionnaires (Additional file 1) both consisted of two parts; the first part intended to evaluate usage and user satisfaction of the app, which was only intended for those in the app user (‘downloaders’) group. The second part was filled out by respondents in both the app user and the non-app user group, and intended to evaluate the effects of having the app, or not, on determinants such as knowledge, perceived severity and susceptibility, self-efficacy, response efficacy and intention to comply with measures to prevent tick bites and LB. The different utilities of the app correspond with these determinants. During the first questionnaire, respondents in the app user group who had only just downloaded the app were asked to take some time to explore the app. Respondents in the app user group who already downloaded the app before participating in this survey were assumed to have familiarized themselves with the app’s content.

To evaluate usage and user satisfaction, app user group respondents were asked to give feedback on the app (Additional file 1: Questionnaire 1). This involved questions about usefulness of different elements of the app, how many times the app was used (if downloaded prior to participation in the survey), and whether one would recommend the app to others. In the second questionnaire (Additional file 1: Questionnaire 2), questions about usage and user satisfaction were asked again to assess the captivating effect of the app. This time the questions were about ongoing usage and user satisfaction since respondents had access to the app for a longer period of time.

For the effectiveness evaluation, the effects of the app on respondents’ knowledge, perceived severity, perceived susceptibility, self-efficacy, response efficacy, and intention regarding the prevention of tick bites and LB were assessed. These determinants were derived from the Protection Motivation Theory (PMT) and the Health Belief Model (HBM) and correspond to our previous evaluation studies of an educational game, leaflet and movie on ticks [15, 16, 20, 21]. Different components of the app are expected to influence the following psychosocial determinants: the information on ticks and LB is expected to influence knowledge and perceived severity, information on checking and removing ticks is expected to influence response efficacy and self-efficacy and intention, and tick radar information is expected to influence perceived susceptibility. The questionnaire was developed further on the basis of an existing questionnaire already used in a study to evaluate the effect of a movie and a leaflet on prevention of tick bites and LB in the Netherlands [22]. In this questionnaire, different psychosocial determinants were based on parts of the PMT and the HBM [20, 21]. The PMT proposes that intention to protect oneself against a health threat is based on the threat appraisal and the coping appraisal, which each depend on two factors. The appraisal of a health threat, which is also part of the HBM, consists of perceived severity (How severe are the consequences of the disease?) and perceived vulnerability (How probable is it that I will contract the disease?). The coping appraisal consists of response efficacy (How effective is the recommended behavior in avoiding the negative consequences?) and self-efficacy (To what extent am I able to perform the recommended behavior successfully?) [23]. To assess whether there was a possible lasting effect of the app on the different determinants, questions to evaluate effectiveness were asked in both the first and the second questionnaire.

In this questionnaire, level of knowledge was measured with eight true/false questions (e.g. ‘Ticks usually fall out of trees to bite.’). The other psychosocial determinants were measured on a 7-point Likert scale, ranging from 1 (totally disagree) to 7 (totally agree). Response efficacy was measured with five questions (e.g. ‘Checking for ticks after every outdoor visit will help prevent LB.’), self-efficacy with five questions (e.g. ‘I am capable of recognizing a tick on my body.’), intention with four questions (e.g. ‘I am planning to check my skin for ticks after every outdoor visit.’), perceived susceptibility with two questions (e.g. ‘How likely do you think it is that you will be diagnosed with LB within the next year?’), and perceived severity with three questions (e.g. ‘If I were to sustain a tick bite, I would be worried about the possible consequences.’). Personal demographics were recorded, including gender, age, and level of education achieved. In addition, questions about everyday use of mobile apps, amount of time spent outdoors (increased risk of tick bites), and having children aged 0–17 years at home (increased tick awareness) were asked.

#### Statistical analyses

Data from questionnaires 1 and 2 were analyzed using SPSS 22 for Windows. To assess user satisfaction, means (standard deviation (SD)) and proportions were used. The study population was described using means (SD) and proportions. Sum scores were calculated for questions on knowledge. If psychosocial items showed sufficient internal consistency, they were measuring the same construct, and were therefore assembled into a single construct. Since Cronbach’s alpha is an inadequate estimate for both validity and reliability, omega was used to assess the internal structure of the items in each scale [24]. Perceived severity was assessed by means of 3 items (Ω = 0.90, 95% CI = 0.88–0.92), perceived susceptibility by means of 2 items (Ω = 0.56, 95% CI = 0.46–0.64), self-efficacy by means of 5 items (Ω = 0.72, 95% CI = 0.67–0.77), response efficacy by means of 5 items (Ω = 0.80, 95% CI = 0.77–0.84), and intention by means of 4 items (Ω = 0.69, 95% CI = 0.63–0.75). The full text of all items can be found in Appendix 1.

After dichotomization of the items on the 7-point scale, odds ratios (ORs) with 95% confidence intervals (CI) were calculated, with a *P* value from a Chi-square test, to find associations between psychosocial determinants and the two groups. When univariate analysis showed significant *p*-values (*p* < 0.05), multivariate logistic regression analyses, adjusted for gender, having children aged 0–17 years at home, and having experience of tick bites, was used to calculate adjusted ORs. Associations between the different determinants were corrected for multiple testing using the Benjamini-Hochberg method [25].

## Results

### Usage of the ‘Tekenbeet’ app according to Google analytics

**Fig. 3:**
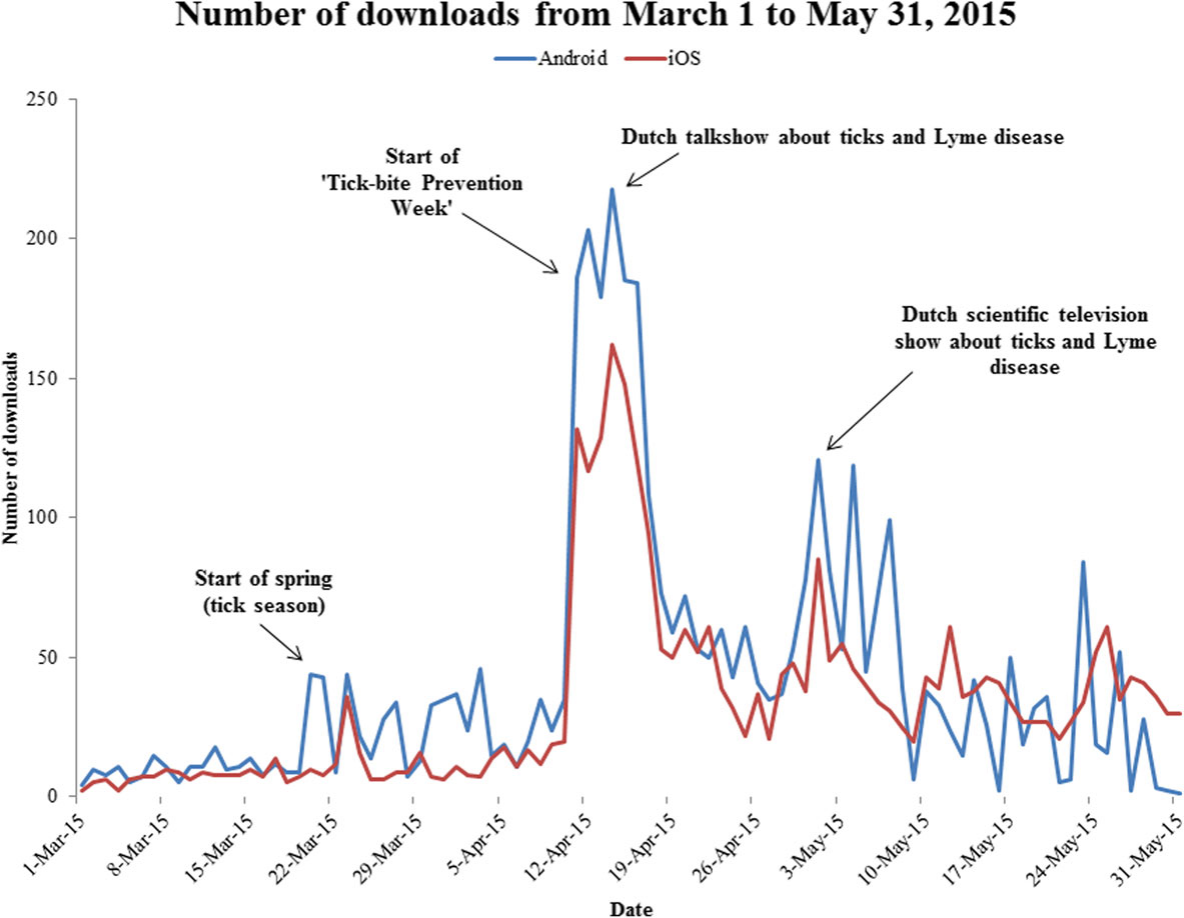
Overview of Android and iOS downloads between March and May 2015

Google Analytics was accessed to gain insight in user statistics. Since the launch in April and July 2014 respectively, until December 1st 2015, the Android version of the app had been downloaded 25,783 times (62.9% of total downloads), and the iOS version had been downloaded 15,221 times (37.1% of total downloads). There were several peak moments when the app was downloaded considerably more frequently. These coincided with certain media events about ticks and tick bites, such as the introduction of the app itself, television shows, ‘Tick Bite Prevention Week’, and tweets and Facebook messages about the app. Figure 3 shows a download timeline between 1st March and 31st May 2015. During the colder months (October–March) when ticks are less active, the amount of downloads was lowest (data not shown).

As of December 1st, 2015, measured from the initial app publication date of April 2014, Google Analytics showed that 67.5% of the Android app users was a returning user, meaning they accessed the app at least twice. Users viewed the tick bite screen most often (this is the start screen of the app), followed by the tick radar, the tick diary screen, and the tick alarm screen. An overview of popularity of the different screens can be found in Table 1.

**Table 1:** An overview of popularity of the different screens

Screen name	Number of screen views	Number of unique screen views	Average time on screen in minutes
Tekenbeet (‘Tick bite’)	93,268	75,734	0:20
Tekenradar (‘Tick radar’)	38,636	29,348	0:54
Tekendagboek (‘Tick diary’)	25,245	13,355	0:39
Tekenalarm (‘Tick alarm’)	24,372	19,312	0:30
De teek (‘Tick info’)	18,921	15,354	0:42
Controleren (‘Tick check’)	16,039	14,146	0:43
Verwijderen (‘Tick removal’)	15,549	13,698	1:00
Ziekte van Lyme (‘Lyme disease’)	12,742	10,511	0:46
Veelgestelde vragen (‘FAQ’)	8759	7587	2:10
Teek herkennen (‘Tick identification’)	3778	3219	0:42
Total	258,238	203,051	0:39

The rating for the ‘Tekenbeet’ app in the Google Play Store (based on 250 ratings, as of December 1st 2015), was 4.1 out of 5. According to the rating information found on the iOS App Store (based on 26 ratings, as of December 1st 2015), its rating was 4+. These numbers provide a measure of popularity/appreciation of the app.

### Usage, user satisfaction and the effects of the ‘Tekenbeet’ app according to the survey

#### Response

**Fig. 4:**
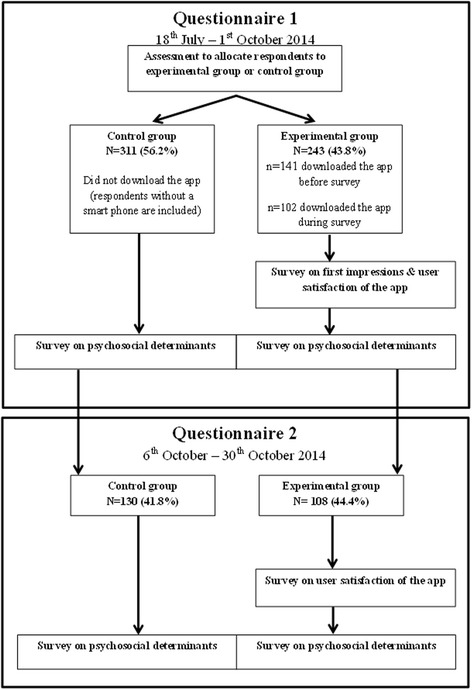
Flowchart depicting response rates of participants in the survey

A total of 812 respondents started filling out the questionnaire, while 258 did not finish the complete questionnaire, resulting in 554 complete questionnaires. Most respondents were recruited during the first few weeks of the inclusion period. Of the 554 respondents, 340 gave details of their e-mail address. On 6th October 2014 they received an invitation e-mail requesting completion of the second questionnaire. A reminder e-mail was sent on 16th October. Seventy percent of the invitees (238 out of 340) completed the second questionnaire (Fig. 4).

#### Respondents

Sixty-three point four percent (*n* = 351/554) of the respondents was female and the mean age was 46.6 years (SD: 13.5). Sixty-two point three percent (*n* = 345/554) had a high educational level (defined as higher education or university), and 33.9% (*n* = 188/554) had children aged 0–17 living at home. Eighty-seven point five percent (*n* = 485/554) had tick bite experience themselves or through someone they knew, whereas 56.5% (*n* = 313/554) had LB experience themselves or trough someone they knew. Twenty point four percent (*n* = 113/554) had a profession that involved working outdoors. Most of the respondents, 88.7% (*n* = 490/546), indicated that in the three months prior to filling out the questionnaire they sometimes or always checked for tick bites following potential exposure.

When comparing the app user group (i.e. those who downloaded the app) to the non-app user group (respondents who did not download the app), the app user group contained significantly more male respondents, more respondents who had children aged 0–17 living at home, and more people who had experienced a tick bite themselves or knew someone who had. Table 2 presents an overview of the demographics and how they were distributed within the two groups.

**Table 2:** Demographics of respondents

Characteristics	Total (*N* = 554)	Non-app user group (*N* = 311)	App user group (*N* = 243)	Odds ratio	95% CI	*P* value
	% (n/N)	% (n/N)	% (n/N)			
Personal data						
Mean age [years (SD)]	46.6 (13.5)	46.4 (14.1)	46.9 (12.6)	–	–	.37
Gender						
Male	36.6 (203/554)	31.5 (98/311)	43.2 (105/243)	Ref.	–	–
Female	63.4 (351/554)	68.5 (213/311)	56.8 (138/243)	0.61	0.43–0.86	.005
Educational level						
Lower education	37.7 (209/554)	38.9 (121/311)	36.2 (88/243)	Ref.	–	–
Higher education	62.3 (345/554)	61.1 (190/311)	63.8 (155/243)	1.12	0.79–1.59	.52
Children (aged 0–17) living at home						
No	66.1 (366/554)	70.7 (220/311)	60.1 (146/243)	Ref.	–	–
Yes	33.9 (188/554)	29.3 (91/311)	39.9 (97/243)	1.60	1.13–2.29	.009
Professional data						
Being outdoors professionally						
No	79.6 (441/554)	79.7 (248/311)	79.4 (193/243)	Ref.	–	–
Yes	20.4 (113/554)	20.3 (63/311)	20.6 (50/243)	1.02	0.67–1.55	.93
Tick/Lyme experience						
Either they themselves or someone they know has incurred a tick bite						
No	12.5 (69/554)	15.1 (47/311)	9.1 (22/243)	Ref.	–	–
Yes	87.5 (485/554)	84.9 (264/311)	90.9 (221/243)	1.79	1.05–3.06	.03
Having had LB themselves or know someone who has						
No	43.5 (241/554)	43.4 (135/311)	43.6 (106/243)	Ref.	–	–
Yes	56.5 (313/554)	56.6 (176/311)	56.4 (137/243)	0.99	0.71–1.39	.96
Past behavior (checked for tick bites in the past three months)^a^						
Never	10.3 (56/546)	12.5 (38/304)	7.4 (18/242)	Ref.	–	–
Sometimes/always	88.7 (490/546)	87.5 (266/304)	92.6 (224/242)	1.78	0.99–3.20	.05

^**a**^Eight people indicated not to have been in green areas in the past three months and were therefore not included in the analyses

#### Usage and user satisfaction of the app

**Fig. 5:**
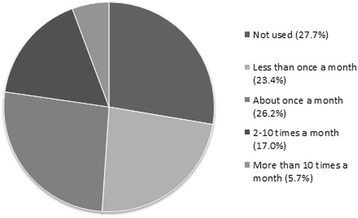
Overview of recent usage of the ‘Tekenbeet’ app

Just over 8 % (8.3%; *n* = 46/554) of the respondents indicated that they did not own an app-enabled mobile phone. Therefore, these respondents were excluded in the questions about app usage and user satisfaction. Forty-five point one percent (*n* = 229/508) of the respondents who owned a mobile phone indicated they were aware of availability of the ‘Tekenbeet’ app and 27.8% (*n* = 141/508) had already downloaded the app prior to the survey. An overview of how often these respondents used the app in the recent past (ranging from one to ten weeks) can be found in Fig. 5. Thirty-one point one percent (*n* = 158/508) were planning to download the app. When these participants were asked to download the app at that moment, 64.6% (*n* = 102/158) of them did so. In total, 43.9% (*n* = 243/554) had the ‘Tekenbeet’ app on their mobile phone at the moment of the first questionnaire.

The mean score on user satisfaction (1–10) was 7.44 (SD 1.22). When asked whether they felt ‘attracted’ to the app, on a scale of 1 (not at all attracted) to 7 (very much attracted), the mean score was 5.62 (SD 1.21). Over 90 % of respondents (90.9%, *n* = 221/243) indicated they would recommend the app to others. Timing of app download - i.e. prior to or during the questionnaire - did not influence these results. Respondents were asked which section of the app they thought was most useful and which part was least useful (see Table 3 for data).

**Table 3:** Usefulness of different sections of the ‘Tekenbeet’ app

Section	Most useful^a^ % (n/N)	Least useful^a^ % (n/N)
Information about removing ticks	39.1 (95/243)	4.1 (10/243)
Tick radar	35.4 (86/243)	20.2 (49/243)
Information about LB	26.3 (64/243)	4.5 (11/243)
Information about checking for ticks	24.7 (60/243)	3.3 (8/243)
Tick diary	24.3 (59/243)	16.5 (40/243)
Information about ticks	22.2 (54/243)	4.5 (11/243)
Tick alarm	22.2 (54/243)	12.3 (30/243)
Frequently asked questions	19.3 (47/243)	5.3 (13/243)

^a^It was possible to indicate multiple sections as most or least useful

Most participants, 78.2% (*n* = 190/243), indicated that they thought information in the app was presented as expected. In questionnaire 2, user satisfaction (range from 1 to 10) mean score was 7.35 (SD 0.96). A near identical score to questionnaire 1 was achieved for recommendation of the app to others in questionnaire 2, at 90.6% (*n* = 87/96). More than half of the respondents in questionnaire 2 (53.1%; *n* = 60/113) indicated that they used the app 1–3 times since they downloaded it.

#### Effects of the app (Additional file 1: Questionnaire 1)

Table 4 presents an overview of the results of the univariate analyses of the determinants according to questionnaire 1; ninety-three point 5 % (*n* = 518/554) of the respondents had a positive intention to check for and remove ticks.

**Table 4:** Univariate and multivariate analyses of determinants in questionnaire 1

Questionnaire 1										
				Univariate analysis			Multivariate analysis *(corrected for gender, having children aged 0–17 years at home, and having experience with a tick bite)*			
Variables, % (n/N)	Total (*N* = 554)	Non-app user group (*N* = 311)	App user group (*N* = 243)	Odds ratio	95% CI	*P* value	Odds ratio	95% CI	*P* value	Transformed *P* value
	% (n/N)	% (n/N)	% (n/N)							
Knowledge (scale range 1–8)										
Low (1–4)	23.5 (130/554)	32.2 (100/311)	12.3 (30/243)	Ref.	–	–	Ref.	–	–	
High (5–8)	76.5 (424/554)	67.8 (211/311)	87.7 (213/243)	3.37	2.15–5.28	<.001	3.21	2.02–5.09	< 0.001	.01
Perceived severity (scale range 1–7)										
Negative (1–4)	53.8 (298/554)	52.1 (162/311)	56.0 (136/243)	Ref.	–	–	Ref.	–	–	
Positive (5–7)	46.2 (256/554)	47.9 (149/311)	44.0 (107/243)	0.86	0.61–1.20	.36	0.94	0.67–1.34	0.743	.74
Perceived susceptibility 1 (scale range 1–7)										
Negative (1–4)	48.0 (266/554)	46.6 (145/311)	49.8 (121/243)	Ref.	–	–	Ref.	–	–	
Positive (5–7)	52.0 (288/554)	53.4 (166/311)	50.2 (122/243)	0.88	0.63–1.23	.46	0.77	0.54–1.09	0.142	.19
Perceived susceptibility 2 (scale range 1–7)										
Negative (1–4)	82.7 (458/554)	80.1 (249/311)	86.0 (209/243)	Ref.	–	–	Ref.	–	–	
Positive (5–7)	17.3 (96/554)	19.9 (62/311)	14.0 (34/243)	0.65	0.41–1.03	.07	0.67	0.42–1.07	0.092	.15
Self-efficacy (scale range 1–7)										
Negative (1–4)	7.6 (42/554)	10.3 (32/311)	4.1 (10/243)	Ref.	–	–	Ref.	–	–	
Positive (5–7)	92.4 (512/554)	89.7 (279/311)	95.9 (233/243)	2.67	1.29–5.55	.01	2.56	1.22–5.37	0.013	.05
Response efficacy (scale range 1–7)										
Negative (1–4)	12.3 (68/554)	14.8 (46/311)	9.1 (22/243)	Ref.	–	–	Ref.	–	–	
Positive (5–7)	87.7 (486/554)	85.2 (265/311)	90.9 (221/243)	1.74	1.02–2.99	.04	1.65	0.96–2.86	0.072	.14
Intention (scale range 1–7)										
Negative (1–4)	6.5 (36/554)	8.7 (27/311)	3.7 (9/243)	Ref.	–	–	Ref.	–	–	
Positive (5–7)	93.5 (518/554)	91.3 (284/311)	96.3 (234/243)	2.47	1.14–5.36	.02	2.67	1.22–5.85	0.014	.04

Respondents in the app user group scored significantly more often high on knowledge compared to respondents in the non-app user group (OR 3.37; 95% CI 2.15–5.28); the same applied to self-efficacy (OR 2.67; 95% CI 1.29–5.55). The app user group more often had a more positive intention to comply with measures to prevent tick bites and LB when compared to the non-app user group (OR 2.47; 95% CI 1.14–5.36); a similar impact was observed for response efficacy (OR 1.74; 95% CI 1.02–2.99). There was no significant difference between the two groups in perceived susceptibility or severity scores.

Multivariate logistic regression analyses showed that gender, having children aged 0–17 years at home, and having experience with a tick bite, had only a very minimal effect on the significance of the relationship between the determinants and the two groups for both intention and knowledge. However, response efficacy and self-efficacy were no longer significantly different in both groups. The results of the multivariate analyses can be found in Table 4.

#### Effects of the app (Additional file 1: Questionnaire 2)

In the second questionnaire, univariate analyses showed no significant differences between the two groups for any determinant. An overview of results in questionnaire 2 can be found in Table 5.

**Table 5:** Univariate analyses of determinants in questionnaire 2

Questionnaire 2						
				Univariate analysis		
Variables	Total (*N* = 238)	Non-app user group (*N* = 130)	App user group (*N* = 108)	Odds ratio	95% CI	*P* value
	% (n/N)	% (n/N)	% (n/N)			
Knowledge (scale range 1–8)^a^						
Low	22.7 (54/238)	26.9 (35/130)	17.6 (19/108)	Ref.	–	–
High	77.3 (184/238)	73.1 (95/130)	82.4 (89/108)	1.73	0.92–3.24	.09
Perceived severity (scale range 1–7)^b^						
Negative	51.4 (119/226)	53.7 (65/121)	51.4 (54/105)	Ref.	–	–
Positive	47.3 (107/226)	46.3 (56/121)	48.6 (51/105)	1.10	0.65–1.85	.731
Perceived susceptibility 1 (scale range 1–7)^b^						
Negative	44.5 (106/238)	45.4 (59/130)	43.5 (47/108)	Ref.	–	–
Positive	55.5 (132/238)	54.6 (70/130)	56.5 (61/108)	1.08	0.65–1.80	.77
Perceived susceptibility 2 (scale range 1–7)^b^						
Negative	87.3 (145/166)	87.4 (76/87)	87.3 (69/79)	Ref.	–	–
Positive	12.7 (21/166)	12.6 (11/87)	12.7 (10/79)	1.00	0.40–2.50	.998
Self-efficacy (scale range 1–7)^b^						
Negative	4.2 (10/238)	5.4 (7/130)	2.8 (3/108)	Ref.	–	–
Positive	95.8 (228/238)	94.6 (123/130)	97.2 (105/108)	1.99	0.50–7.90	.32
Response efficacy (scale range 1–7)^b^						
Negative	8.8 (21/238)	9.2 (12/130)	8.3 (9/108)	Ref.	–	–
Positive	91.2 (217/238)	90.8 (118/130)	91.7 (99/108)	1.12	0.45–2.76	.81
Intention (scale range 1–7)^b^						
Negative	5.5 (13/238)	6.9 (9/130)	3.7 (4/108)	Ref.	–	–
Positive	94.5 (225/238)	93.1 (121/130)	96.3 (104/108)	1.93	0.60–6.46	.28

^a^score 1–4 = negative, score 5–8 = positive

^b^score 1–4 = negative, score 5–7 = positive

#### Results of questionnaire 1 compared to questionnaire 2

By comparing the results of questionnaire 1 to the results of questionnaire 2, it shows response efficacy was higher during questionnaire 2. This was the case in the app user group (5.88 versus 5.67, for questionnaires 2 and 1 respectively), as well as in the non-app user group (5.68 versus 5.46, for questionnaires 2 and 1, respectively). Results can be found in Tables 6 and 7.

**Table 6:** Determinants in questionnaire 1 and 2 for the app user group^a^

	App user group		
	Questionnaire 1 (*N* = 108)	Questionnaire 2 (*N* = 108)	*P* value
	Mean (SD)	Mean (SD)	
Knowledge (scale range 1–8)	6.05 (1.44)	6.04 (1.50)	.94
Perceived severity (scale range 1–7)	3.99 (1.63)	3.95 (1.56)	.69
Perceived susceptibility 1 (scale range 1–7)	4.65 (1.74)	4.63 (1.67)	.87
Perceived susceptibility 2 (scale range 1–7)	2.65 (1.39)	2.82 (1.46)	.14
Self-efficacy (scale range 1–7)	5.94 (0.81)	6.04 (0.79)	.12
Response efficacy (scale range 1–7)	5.67 (1.20)	5.88 (1.09)	.03
Intention (scale range 1–7)	6.14 (0.96)	6.27 (0.87)	.06

^a^Only data of respondents who completed both questionnaires have been taken into account

**Table 7:** Determinants in questionnaire 1 and 2 for the non-app user group^a^

	Non-app user group		
	Questionnaire 1 (*N* = 130)	Questionnaire 2 (*N* = 130)	*P* value
	Mean (SD)	Mean (SD)	
Knowledge (scale range 1–8)	5.29 (1.61)	5.40 (1.52)	.39
Perceived severity (scale range 1–7)	4.04 (1.66)	4.04 (1.66)	.98
Perceived susceptibility 1 (scale range 1–7)	4.82 (1.78)	4.81 (1.65)	.90
Perceived susceptibility 2 (scale range 1–7)	2.85 (1.58)	2.71 (1.49)	.32
Self-efficacy (scale range 1–7)	5.69 (1.14)	5.79 (0.97)	.14
Response efficacy (scale range 1–7)	5.46 (1.24)	5.68 (1.17)	.02
Intention (scale range 1–7)	6.01 (1.10)	6.06 (1.00)	.41

^a^Only data of respondents who completed both questionnaires have been taken into account

## Discussion

The aim of this study was to evaluate the usage and user satisfaction of the ‘Tekenbeet’ app and to investigate whether the app affects users’ knowledge, perceived severity, perceived susceptibility, self-efficacy, response efficacy, current behavior and intention to comply with measures to prevent tick bites and LB.

### Usage

Since its launch two years ago, the ‘Tekenbeet’ app has been downloaded over 41,000 times. This number is high when compared with other non-tick related apps published by the Dutch government. According to data obtained from Google Analytics, more than half of the downloads was by a returning user, meaning they accessed the app at least twice. The survey shows almost 50% of the respondents used the app at least once a month, which supports the Google Analytics data. Compared to other apps, the ‘Tekenbeet’ app is a so-called ‘seasonal app’; customers tend to use it during the tick season between March and October, when there is a more acute need for information about ticks and LB, which is reflected in the increased downloads during the tick season.

There are two other Dutch apps available in app stores that focus on ticks and tick bites: ‘Teek!’ (Dutch translation of ‘Tick!’) is an app developed by the Public Health Service Zeeland; and ‘Teek Away’ (Dutch translation of ‘Tick Away’, a wordplay on the English word takeaway) produced by the organization ‘Nature and Environment Overijssel’. Both apps are rated 3.5 out of 5 by users on the Google Play Store and each has been downloaded between 1000 and 5000 times (data Google Play Store).

In comparison with these two other Dutch tick-focused apps to the ‘Tekenbeet’ app, ‘Tekenbeet’ offers a more interactive experience, such as instruction videos and a tick alarm that can be set to a tick-related activity by the end-user. More importantly, unlike the other apps, the ‘Tekenbeet’ app also functions when there is no Internet connectivity and can therefore be used anytime and anywhere. This can be particularly useful for outdoor activities when there is a lack of Internet connection and potentially a high risk of incurring a tick bite.

### User satisfaction

Our survey data shows that most users felt ‘attracted’ to the app and would recommend it to others; with a mean score of 7.44 on a scale of 1 to 10, user satisfaction was high. This is reflected by the rating of the app in the Google Play Store. This is slightly higher compared to the average medical app [26]. It is clear there were some conflicting opinions regarding its most useful content. Google Analytics shows that the tick radar had the most screen views. However, in the survey the tick radar was appreciated and not appreciated in near equal measures. The appreciation suggests that people may find it useful to make an assessment of the tick risk based on the tick radar data. From the comments made by survey participants, we deduct that disapproval can be explained by the fact that you cannot zoom in on the tick radar map (it can only give a national overview), and it therefore for some lacks a sufficiently detailed display of activity in a specific geographic region that the user wishes to explore. In the survey, the screen with information about removing ticks was rated ‘most useful’, but as per Google Analytics, this screen was 6th in terms of most screen views. This may be explained by the fact that the information on this screen is only useful in the event of an actual tick bite.

### Effects

Based on our survey results, we conclude that the ‘Tekenbeet’ app is an effective educational tool to increase public knowledge, and to meet the intention to improve preventive behavior regarding ticks and tick bites. Knowledge was high in both the downloader and non-downloader groups, which is consistent with other findings concerning other media used for educational purposes [22], although app users were slightly more knowledgeable. This might be explained by the fact that they could look up certain answers on the knowledge questions in the app, or because they were aware of the risks of tick bites and were therefore keener to download the app. However, respondents who did not download the app could potentially have looked up the answers too via different sources such as websites. The outcomes for the knowledge aspect might explain why intention to take preventive measures was also higher in the app user group, since knowledge is a known determinant that has a positive influence on intention [14].

Our previous research has shown that a movie and leaflet for prevention of tick bites and LB are effective educational tools for improving knowledge, and that these types of media help to increase the intention - at least in the short-term - to take preventative measures concerning tick bites and LB [22]. Unfortunately, these improvements in knowledge and intention could no longer be detected during the second measurement, which meant an enduring effect of information provided through a leaflet or movie was lacking. To consult a leaflet or movie as a consumer, you need to have the leaflet at hand or know where to find the movie online. In contrast, the ‘Tekenbeet’ app is readily available once downloaded (with only the tick radar requiring live Internet connection), which may help users to keep consulting it repeatedly over a longer period. This could mean it can have a ‘perpetuating short-term effect’ rather than the one-off short-term effect we have seen with the movie and the leaflet.

When comparing the results of questionnaire 1 to the results of questionnaire 2, response efficacy seems to have increased in both the app user group and the non-app user group. Response efficacy refers to a person’s belief as to whether recommended actions (e.g. removing/checking for ticks) will help to avoid the threat (e.g. a tick bite/Lyme disease). This can be an effect of the questionnaire itself. Possibly respondents learned about the importance of removing/ checking for ticks by filling out the questionnaires, and therefore scored higher on response efficacy.

It is a challenge to also reach people who are less intrinsically interested in this subject and make them aware of the availability of the app. One option would be to promote the app on notice boards at places with a higher tick bite risk, such as visitor centers in national parks, camp sites, or at outdoor festivals. Furthermore, promoting the app in other educational materials, such as the RIVM’s website, leaflet and the online movie about ticks and LB, or in publications by for instance primary care, may increase awareness of the availability of the app. After all, an app store may not be the natural place one looks for information on ticks.

#### Limitations and future research

We were pleasantly surprised to learn that almost half of the respondents indicated that they were aware of the availability of the ‘Tekenbeet’ app before participating in the survey. An invitation to participate in the survey was posted on websites with a special interest in nature or ticks and LB, which was also the place where the app was promoted in the months prior to the survey. This may explain why so many respondents were aware of the availability of the app. However, being aware of the availability of the app due to promotion does not necessarily mean someone is willing to download the app. This can be explained by many reasons such as not having a smartphone or simply not being interested in having an app on this subject. Furthermore, almost a third of the respondents who started filling out questionnaire 1 did not complete it, most of them quit after only one or two questions. These factors may have introduced selection bias, but due to this kind of inclusion method, a non-response study was not possible. This recruitment method was chosen to increase the likelihood that native tick-interested people participated in the study, and only native app-interested people would fill out the questions about the app, since these people are precisely the target group of the mobile app. Clearly, the recruitment method used in this study does not provide a representative sample of the Dutch population. However, we were mainly interested in the usage and the effects of the app on the different determinants of people who, in real life, would also use this app. To reduce selection bias, in the multivariate analyses we corrected for gender, having children aged 0–17 years at home, and having experience of tick bites. In the study population there was an overrepresentation of women and people with a higher education level. However, this phenomenon is seen more often when respondents are recruited through websites or social media [27].

People had the option to download the app if they were willing to do so. They were not divided into two groups beforehand, and this may have contributed to selection bias. However, in this study, we were mostly interested in respondents that showed an unforced interest in ticks/LB and/or in downloading the app.

At the end of each questionnaire, respondents could leave comments. This resulted in some useful recommendations to improve its user-friendliness. For example, the inclusion of a functionality to make it possible to send tick diary data to someone else if approved by the user, such as parents or one’s general practitioner. This could be an improvement, especially for teachers or supervisors at children’s nature clubs. Furthermore, the tick radar function in the app was considered ‘least useful’ by 20.2% of respondents, mostly due to the unavailability of a zoom function. These feedback results may be acted upon to make improvements to future updated versions of the app. The introduction of a zoom option for the tick radar is currently being considered.

Future research might be helpful to determine the effect of the app even more. As mentioned before, it is a challenge to reach people who are less intrinsically interested in this subject. Research on how to reach a less engaged and motivated population could be beneficial to achieve the apps’ full potential. Furthermore, using a baseline evaluation might clarify even better how much the application improves knowledge compared to using non-app users as a proxy as was the case in this study.

Finally, a follow-up study could be helpful to determine whether the intention to apply tick prevention measures actually leads to increased tick preventing behavior. However, there is only a limited timeframe in which ticks are active, meaning a follow-up study has to take place a year later.

After such a period, most respondents have probably already forgotten about if and when they were outdoors and if they checked for ticks. Therefore, long periods between measurements would probably not measure the influence of the app anymore and result in recall bias. This can be solved by measurements in quicker succession. Even so, participants in the non-app user group would have the opportunity to download the app in the meantime, which may affect the results of the study.

## Conclusion

In conclusion, the ‘Tekenbeet’ app is a frequently used and well-appreciated educational tool to increase knowledge concerning ticks and tick bites for a large group of the intrinsically motivated population. It appears to be a useful addition to the current range of educational tools, and helps to improve self-efficacy to perform preventive measures (check for and remove ticks), and the user’s intention to apply these measures. The difference in increase in knowledge and intention between the app user and the non-app user group does seem to diminish over time. However, one of the advantages of mHealth is that it can be used anytime and anywhere the information is needed, which may reduce the need for long-term knowledge retention. The use of smartphones and apps is now commonplace; the ‘Tekenbeet’ app feeds into this trend and offers a modern day interactive alternative to other educational tools.

## Appendix

**Table 8:** Internal consistency of different psychosocial determinants, and their corresponding items

	Nr. of items	Internal consistency (Omega_total_)
Perceived severity 1. If I were to sustain a tick bite, I would be worried about the possible consequences. 2. If I were to sustain a tick bite, I would expect this to have a serious impact on my health. 3. If I were to sustain a tick bite, I would expect to suffer from the consequences for quite a long time.	3	Ω=.90
Perceived susceptibility 1. How do you assess the chance that you will be bitten by a tick in the next year? 2. How likely do you think it is that you will be diagnosed with Lyme Disease in the next year?	2	Ω=.56
Self-efficacy 1. I am capable of recognizing a tick on my body. 2. I am capable of checking my skin for tick bites after I have ventured outdoors. 3. I am capable of removing a tick from my skin using pointed tweezers (or a different kind of tick remover). 4. I am capable of writing down place and date that the tick bite occurred. 5. I am capable to visit a GP if a tick has been on my body for over 24 hours.	5	Ω=.72
Response efficacy 1. Recognizing a tick when checking your body will help preventing Lyme disease. 2. Checking for ticks after every outdoor venture will help preventing Lyme disease. 3. Removing a tick immediately using pointed tweezers (or a different kind of tick remover) will help prevent Lyme disease. 4. Writing down place and date when the tick bite occurred will help prevent Lyme disease. 5. Visiting a GP if a tick has been stuck on the skin for over 24 hours will help prevent Lyme disease.	5	Ω=.80
Intention 1. I am planning to check my skin for ticks after every outdoor venture. 2. If I discover a tick bite on my body, I am planning to remove the tick immediately. 3. If I discover a tick bite on my body, I am planning to note the place of the bite and the date it occurred. 4. I am planning to visit a GP if a tick has been stuck on my skin for over 24 hours.	4	Ω=.69

## Additional file

Additional file 1: Questionnaires (DOCX 38 kb)

## Abbreviations

EM: erythema migrans
HBM: Health Belief Model
LB: Lyme borreliosis
PMT: Protection Motivation Theory
RIVM: Dutch National Institute for Public Health and the Environment
