# Supplementary material for: Prevention of tick bites: an evaluation of a smartphone app
Source: BMC Infect Dis. 2017 Dec 4;17:744. doi: 10.1186/s12879-017-2836-4 (PMC5716000; doi:10.1186/s12879-017-2836-4)
Supplement: Additional file 1: — Questionnaires (DOCX 38 kb) [file 12879_2017_2836_MOESM1_ESM.docx]

**Additional file 1: Questionnaires**

**Questionnaire 1**

**Research on ticks and Lyme disease**

**Part 1: questions regarding your knowledge on ticks and Lyme disease.**

1. You are now going to see eight statements. Please read them and indicate if you think the statement is ‘true’ or ‘false’. If you do not know the answer please mark ‘I don’t know’.

|  | true | false | I don’t know |
| --- | --- | --- | --- |
| A tick usually falls from a tree in order to bite. |  |  |  |
| You can remove a tick by pulling it directly upwards with pointed tweezers. |  |  |  |
| A tick bite always makes you ill. |  |  |  |
| Ticks mostly bite in so-called ‘warm areas’, e.g. armpits, groin, or behind the knee. |  |  |  |
| After removing a tick, you should monitor your health for up to a week maximum. |  |  |  |
| If part of a tick remains in the skin, this is dangerous. |  |  |  |
| In Lyme disease a red ring usually appears on the skin. |  |  |  |
| Over half of all ticks is infected with the bacterium responsible for Lyme disease. |  |  |  |

**Part 2: questions regarding app use.**

**Below, we ask questions about your experience regarding app use in general and the RIVM app ‘Tekenbeet’ in particular. Please note: this study only focusses on the RIVM app ‘Tekenbeet’, and does not focus on other apps regarding ticks, such as ‘Teek’ or ‘Teek away’.**

2. Which brand of mobile phone do you use?

- No mobile phone 🡪 go to question 23
- iPhone
- Samsung
- HTC
- LG
- Other: ………

3. Do you ever download apps on your mobile phone (excluding messaging services such as WhatsApp Messenger, e-mail, and calendar)?

- Yes
- No 🡪 go to question 5

4. How often do you download apps on your mobile phone (excluding messaging services such as WhatsApp Messenger, e-mail and calendar)?

- Less than once a month
- 1-2 times in 6 months
- 3-5 times in 6 months
- 1-3 times a month
- 1-2 times a week
- 3-4 times a week
- 5-6 times a week
- Every day

5. How often do you use apps on your mobile phone (excluding messaging services such as WhatsApp Messenger, e-mail and calendar)?

- Less than once a month
- 1-2 times in 6 months
- 3-5 times in 6 months
- 1-3 times a month
- 1-2 times a week
- 3-4 times a week
- 5-6 times a week
- Every day

6. Are you familiar with the RIVM app ‘Tekenbeet’ about ticks and Lyme disease?

- Yes
- No

7. Did you download the app ‘Tekenbeet’ on your mobile phone, or are you planning to do so?

- No, I did not download or plan to download the app 🡪 go to question 24
- Yes, I am planning to download the app
- Yes, I downloaded the app

8. Why did you not download the app ‘Tekenbeet’ or do you not intend to do so?

……………..🡪 go to question 24

9. Did you use the app ‘Tekenbeet’ in the recent past?

- No
- Less than once a month
- About once a month
- 2-10 times a month
- More than 10 times a month

**We ask you to download the app ‘Tekenbeet’ on your phone. You can find this app in the Google Play Store or in the App Store.**

10. Did you manage to download the app ‘Tekenbeet’?

- Yes
- No 🡪 go to question 24

**Part 3: Questions regarding the content of the app ‘Tekenbeet’.**

**Below we ask you for your opinion regarding the app ‘Tekenbeet.**

11. How would you rate the app (1-10)?

- 1
- 2
- 3
- 4
- 5
- 6
- 7
- 8
- 9
- 10

12.

|  | 1 – not at all | 2 | 3 | 4 | 5 | 6 | 7 – very much |
| --- | --- | --- | --- | --- | --- | --- | --- |
| Do you feel addressed by the app |  |  |  |  |  |  |  |

13. Would you recommend the app to friends or family?

- Yes 🡪 go to question 14
- No 🡪 go to question 15

14. Why would you recommend the app to friends or family?

……………..🡪 go to question 16

15. Why would you not recommend the app to friends or family?

……………..

16. What would you like to change about the app?

……………..

17. Which part(s) of the app do you consider most useful? (more than one answer is possible)

- All parts are evenly useful
- ‘the tick’
- ‘tick radar’
- ‘to control’
- ‘to remove’
- ‘Lyme disease’
- ‘tick alert’
- ‘frequently asked questions’
- ‘tick diary’

18. Which part(s) of the app do you consider least useful? (more than one answer is possible)

- All parts are evenly useful
- ‘the tick’
- ‘tick radar’
- ‘to control’
- ‘to remove’
- ‘Lyme disease’
- ‘tick alert’
- ‘frequently asked questions’
- ‘tick diary’

19. Does the app provide the information you are looking for?

- Yes 🡪 go to question 21
- Partially
- No

20. What kind of information would you like to find in the app?

……………..

21. In what manner would you prefer to receive information about ticks and Lyme disease?

- Through a leaflet
- Through a website
- Through an app
- Through a movie
- Other: …….

22. You are now going to see eight statements. Please read them and indicate if you think the statement is ‘true’ or ‘false’. If you do not know the answer please mark ‘I don’t know’. We are aware you have already answered these questions in the beginning of the questionnaire, but it is important that you answer them once again.

|  | true | false | I don’t know |
| --- | --- | --- | --- |
| A tick usually falls from a tree in order to bite. |  |  |  |
| You can remove a tick by pulling it directly upwards with pointed tweezers. |  |  |  |
| A tick bite always makes you ill. |  |  |  |
| Ticks mostly bite in so-called ‘warm areas’, e.g. armpits, groin, or behind the knee. |  |  |  |
| After removing a tick, you should monitor your health for up to a week. |  |  |  |
| If part of a tick remains in the skin, this is dangerous. |  |  |  |
| In Lyme disease a red ring usually appears on the skin. |  |  |  |
| Over half of all ticks is infected with the bacterium responsible for Lyme disease. |  |  |  |

**Part 4: Questions regarding your personal situation.**

**Below, we will ask you some questions regarding your personal and family situation.**

23. What is your year of birth?

………………

24. What is your gender?

- Male
- Female

25. What is the highest level of education you have completed?

- None (did not finish primary school)
- Primary school (basisschool, special-needs primary school)
- Lower vocational education or Prepratory Vocational Education (e.g. Ambachtsschool, Huishoudschool, LTS, LEAO, LHNO, VMBO)
- Secondary school (e.g. MAVO, (M)ULO, MBO-kort, VMBO-t)
- Secondary vocational education (e.g. training programs for baker or hairdresser, MBO-lang, MTS, UTS, MEAO, BOL, BBL, INAS)
- Higher secondary education or preparatory scientific education (e.g. HAVO, VWO, Atheneum, Gymnasium, HBS, MMS)
- Higher professional education (e.g. kweekschool, HBO, HTS, HEAO, HBO-V, kandidaats wetenschappelijk onderwijs)
- Academic education (university)
- Other, ……….

26. Do you own a dog or a cat?

- No
- Yes, (a) dog(s)
- Yes, (a) cat(s)
- Yes, both

27. Do you have children living at your home (age 0-17)?

- Yes
- No

28. How often are your kids active in green spaces?

*When we talk about green spaces we mean being outdoors in nature areas such as woods, heathland, dunes or a park.*

- Less than once a month 🡪 go to question 32
- Every month
- Every week 🡪 go to question 30
- Daily 🡪 go to question 31

29. On a monthly basis, how many hours does your child on average spend in green spaces?

*When we talk about green spaces we mean being outdoors in nature areas such as woods, heathland, dunes or a park.*

……………..🡪 go to question 32

30. On a weekly basis, how many hours does your child on average spend in green spaces?

*When we talk about green spaces we mean being outdoors in nature areas such as woods, heathland, dunes or a park.*

……………..🡪 go to question 32

31. On a daily basis, how many hours does your child on average spend in green spaces?

*When we talk about green spaces we mean being outdoors in nature areas such as woods, heathland, dunes or a park.*

……………..

32. How often are you active in green spaces?

*When we talk about green spaces we mean being outdoors in nature areas such as woods, heathland, dunes or a park.*

- Less than once a month 🡪 go to question 36
- Every month
- Every week 🡪 go to question 34
- Daily 🡪 go to question 35

33. On a monthly basis, how many hours do you on average spend in green spaces?

*When we talk about green spaces we mean being outdoors in nature areas such as woods, heathland, dunes or a park.*

……………..🡪 go to question 36

34. On a weekly basis, how many hours do you on average spend in green spaces?

*When we talk about green spaces we mean being outdoors in nature areas such as woods, heathland, dunes or a park.*

……………..🡪 go to question 36

35. On a daily basis, how many hours do you on average spend in green spaces?

*When we talk about green spaces we mean being outdoors in nature areas such as woods, heathland, dunes or a park.*

……………..

36. Does your job involve you actively working in green spaces? (e.g. forester or gardener)

*When we talk about green spaces we mean being outdoors in nature areas such as woods, heathland, dunes or a park.*

- Yes
- No 🡪 go to question 41

37. On average, how much time do you spend working in green spaces?

*When we talk about green spaces we mean being outdoors in nature areas such as woods, heathland, dunes or a park.*

- Less than once a month 🡪 go to question 41
- Every month
- Every week 🡪 go to question 39
- Daily 🡪 go to question 40

38. On a monthly basis, how many hours do you on average spend in green spaces professionally?

*When we talk about green spaces we mean being outdoors in nature areas such as woods, heathland, dunes or a park.*

……………..🡪 go to question 41

39. On a weekly basis, how many hours do you on average spend in green spaces professionally?

*When we talk about green spaces we mean being outdoors in nature areas such as woods, heathland, dunes or a park.*

……………..🡪 go to question 41

40. On a daily basis, how many hours do you on average spend in green spaces professionally?

*When we talk about green spaces we mean being outdoors in nature areas such as woods, heathland, dunes or a park.*

……………..

**Part 5: questions regarding ticks and Lyme disease**

**Below, we will ask you questions regarding ticks and Lyme disease.**

41. Have you or anyone in your direct social network (such as children, partner, family, friends) ever had one or more tick bites?

- Yes
- No
- I can’t remember

42. Have you or anyone in your direct social network (such as children, partner, family, friends) ever had Lyme disease?

- Yes
- No 🡪 go to question 44
- I can’t remember 🡪 go to question 44

43. Who diagnosed Lyme disease? (In case you know more cases of Lyme disease, assume the first case that comes to mind)

- General practitioner
- A medical specialist
- Other, ………….
- I don’t know

44. How often over the past year have you checked yourself or someone else for tick bites after visiting a green space?

*When we talk about green spaces we mean being outdoors in nature areas such as woods, heathland, dunes or a park.*

- Never after visiting a green space
- Sometimes after visiting a green space
- Every time after visiting a green space
- I have not visited a green space

45. How often over the past year have you immediately removed a tick from your own or someone else’s skin after discovering a tick bite?

*When we talk about green spaces we mean being outdoors in nature areas such as woods, heathland, dunes or a park.*

- Never after visiting a green space
- Sometimes after visiting a green space
- Every time after visiting a green space
- I have not discovered a tick bite

46. Imagine that you are going to take one of the precautions below to prevent getting Lyme disease. From a practical point of view, to what extent do you think that you would really succeed in taking these precautions?

*When we talk about green spaces we mean being outdoors in nature areas such as woods, heathland, dunes or a park.*

|  | 1 – strongly disagree | 2 | 3 | 4 | 5 | 6 | 7 – strongly agree |
| --- | --- | --- | --- | --- | --- | --- | --- |
| I would be able to recognise a tick on my body. |  |  |  |  |  |  |  |
| I would be able to check my body for tick bites after every trip to a green space. |  |  |  |  |  |  |  |
| I would be able to remove a tick immediately with pointed tweezers (or other type of tick remover). |  |  |  |  |  |  |  |
| I would be able to note down the place of the tick bite on my body and the date. |  |  |  |  |  |  |  |
| I would be able to go to the GP if I had a tick on my skin for over 24 hours. |  |  |  |  |  |  |  |

47. The following statements concern measures to prevent Lyme disease. Please indicate how much you think that these measures would help.

*When we talk about green spaces we mean being outdoors in nature areas such as woods, heathland, dunes or a park.*

|  | 1 – strongly disagree | 2 | 3 | 4 | 5 | 6 | 7 – strongly agree |
| --- | --- | --- | --- | --- | --- | --- | --- |
| Recognising a tick when checking the body helps to prevent Lyme disease |  |  |  |  |  |  |  |
| Checking for ticks after every visit to a green space helps to prevent Lyme disease. |  |  |  |  |  |  |  |
| Removing a tick immediately with pointed tweezers or other type of tick remover helps to prevent Lyme disease. |  |  |  |  |  |  |  |
| Noting down the place and date of the tick bite helps to prevent Lyme disease. |  |  |  |  |  |  |  |
| Going to the GP if you have had a tick on your skin for longer than 24 hours helps to prevent Lyme disease. |  |  |  |  |  |  |  |

48. Imagine that you would want to go to an area where there could be ticks (‘green spaces’). Please indicate below what you intend to do.

*When we talk about green spaces we mean being outdoors in nature areas such as woods, heathland, dunes or a park.*

|  | 1 – strongly disagree | 2 | 3 | 4 | 5 | 6 | 7 – strongly agree |
| --- | --- | --- | --- | --- | --- | --- | --- |
| I intend to check my body for ticks after every visit to a green space. |  |  |  |  |  |  |  |
| If I find a tick on my body I intend to remove it immediately. |  |  |  |  |  |  |  |
| If I find a tick on my or someone else’s body I intend to write down the place of the bite on the body and the date. |  |  |  |  |  |  |  |
| If I have a tick on my skin for more than 24 hours I plan to go to the GP. |  |  |  |  |  |  |  |

49. The following questions concern your expectations regarding tick bites in the coming year.

|  | 1 – very low | 2 | 3 | 4 | 5 | 6 | 7 – very high |
| --- | --- | --- | --- | --- | --- | --- | --- |
| In your opinion, how big is the risk that you will be bitten by a tick in the coming year? |  |  |  |  |  |  |  |
| In your opinion, how big is the risk that you will get Lyme disease in the coming year? |  |  |  |  |  |  |  |

50. The following questions concern your expectations regarding the consequences of tick bites.

|  | 1 – strongly disagree | 2 | 3 | 4 | 5 | 6 | 7 – strongly agree |
| --- | --- | --- | --- | --- | --- | --- | --- |
| If I would have a tick bite, I would worry about the possible consequences. |  |  |  |  |  |  |  |
| If I get a tick bite, I expect serious health consequences. |  |  |  |  |  |  |  |
| If I would have a tick bite, I expect long term consequences. |  |  |  |  |  |  |  |
| I am frightened of ticks or Lyme disease by all the media attention around this subject. |  |  |  |  |  |  |  |

51. Do you have any comments?

……………..

**Thank you for filling in the questionnaire! We would like to ask you to fill in a second (short) questionnaire again within 2-3 months. If you are willing to participate in the second questionnaire, you can leave your e-mail address below. Among participants filling out both questionnaires, 50 gift certificates (10 Euro each) will be raffled. Off course you may use the app as often as you like.**

**E-mail address :**

**Questionnaire 2**

**Research on ticks and Lyme disease**

**Questionnaire 2 contained some of the questions that were also asked during questionnaire 1 (11, 13, 14, 15, 16, 21, 22, 41, 42, 43, 44, 45, 47, 46, 48, 49, 50). Questions concerning behavior in the past year in questionnaire 1 were replaced by questions concerning behavior in the past three months in questionnaire 2. Questions regarding use of the app ‘Tekenbeet’ were excluded for the control group.**

In addition, the following questions were added to questionnaire 2 :

1. Are you familiar with the RIVM app ‘Tekenbeet’ about ticks and Lyme disease?

- No
- Yes, but I did not download the app
- Yes, I downloaded the app

2. When did you download the RIVM app ‘Tekenbeet’

- About 1 day ago
- About 1 week ago
- About 2 weeks ago
- About 3 weeks ago
- About 4 weeks ago
- Longer than 4 weeks ago

3. Did you use the RIVM app ‘Tekenbeet’ after you downloaded it?

- No
- Yes, 1-3 times
- Yes, 4-6 times
- Yes, 7-9 times
- Yes, more than 9 times

4. How would you rate the RIVM app ‘Tekenbeet’(1-10)?

- 1
- 2
- 3
- 4
- 5
- 6
- 7
- 8
- 9
- 10

5. Do you currently have Lyme disease?

- Yes
- No
